# Tensile Modeling PVC Gels for Electrohydraulic Actuators

**DOI:** 10.3390/polym17192641

**Published:** 2025-09-30

**Authors:** John Albert Faccinto, Jongcheol Lee, Kwang J. Kim

**Affiliations:** Department of Mechanical Engineering, University of Nevada, Las Vegas, NV 89154, USA; john.faccinto@unlv.edu (J.A.F.); jongcheol.lee@unlv.edu (J.L.)

**Keywords:** EPIC actuator, PVC gel tensile modeling, dielectric elastomer actuator, elastic modulus, Poisson’s ratio, hyperelastic modeling, PVC modeling, PVC-DBA

## Abstract

Polyvinyl chloride (PVC)-dibutyl adipate (DBA) gels are a fascinating dielectric elastomer actuator showing promise in soft robotics. When actuated with high voltages, the gel deforms towards the anode. A recent application of PVC gels in electrohydraulic actuators motivates elastic and hyperelastic constitutive relationships for tensile loading modes. PVC gels with plasticizer-to-polymer weight ratios of 2:1, 4:1, 6:1, and 8:1 *w*/*w* were evaluated. PVC gels exhibit a linear elastic region up to 25% strain. The elastic modulus decreased with increasing plasticizer content from 288.8 kPa, 56.1 kPa, 24.7 kPa, to 11 kPa. Poisson’s ratio also decreased with increasing plasticizer content from 0.42, 0.43, 0.39, to 0.35. We suggest that the decrease in polymer concentration facilitates a weakly interconnected polymer network susceptible to chain slippage that hinders the network response, thus lowering Poisson’s ratio. Our work suggests that PVC gels can be treated as isotropic and incompressible for large strains and hyperelastic modeling; however, highly plasticized gels tend to act less incompressible at small strains. The power scaling law between the elastic modulus and plasticizer weight ratio showed high agreement, making the elastic modulus deterministic for any plasticizer content. The Neo–Hookean, Mooney–Rivlin, Yeoh, Gent, Ogden, and extended tube hyperelastic constitutive models are investigated. The Yeoh model shows the highest feasibility when evaluated up to 3.5 stretch, showing a maximum normalized root-mean-square-error of 6.85%. Together, these findings establish a constitutive basis for PVC-DBA gels, incorporating small strain elasticity, large strain non-linear behavior, and network analysis while providing suggestive insight into the network structure required for accurately modeling the EPIC.

## 1. Introduction

Polyvinyl chloride (PVC) gels are a promising class of dielectric elastomer actuators (DEAs) possessing a unique actuation mechanism known as anodophilic attraction or creep deformation towards the anode. First reported by the Harai group [1], this mechanism originates from polymer–solvent interactions coupled with variations in space charge density when an external electric field is applied. Charges carried by the plasticizer form a saturated plasticizer layer near the anode electrode interface. This localized enrichment reduces the modulus in the affected region, thereby enabling enhanced electrostatic attraction towards the anode that creates anodophilic deformation [2,3,4]. Many applications and systems have been developed using this DEA. Li et al. have applied PVC gels to artificial muscles by creating an exoskeleton used to improve gait in older individuals with muscular atrophy [5,6,7,8]. Their wearable device utilizes deactivation to generate force for the offending leg. In a similar process, PVC gels have been used in rotary brakes. In the event of power loss, relaxation back to the starting configuration applies to a brake pad to stop a rotor [9,10]. Since the discovery of bending actuation, several contractual gripping actuators have been developed [11,12,13] among many other actuators.

Recently, PVC gels have shown promise in electrohydraulic actuation when researchers at KAIST developed the electrohydraulic actuator powered by induced interfacial charges (EPICs) [14]. This novel approach is reminiscent of hydraulically amplified self-healing electrostatic (HASEL) actuators which use Maxwell pressure to compress an elastomeric shell containing dielectric fluid. Fluid displacement pressurizes non-electrode regions creating displacement and force output [15,16,17,18,19]. The EPIC diverges from HASELs by using PVC film in a round rigid frame that contains dielectric (DE) fluid. Anodophilic attraction zips the gel onto the anode attached to the frame, generating hydraulic pressure and monodirectional hydraulic actuation. Since the PVC gels are softer than the elastomers in HASELs, such as BOPP and Ecoflex 00-30 [20], less energy is expended in material stretching; therefore, large displacements can be achieved at voltages around 3 kV [21]. The compliant electrodes allow the gel to zip which has been shown to enhance actuation in HASELs [22].

The present work investigates elastic and hyperelastic tensile constitutive relationships of PVC gels made with DBA plasticizer. A primary objective in developing these relationships is to describe the EPIC dominant mechanical response during actuation which is biaxial tension from spherical deformation. Studies have analyzed tensile properties of PVC gels with various plasticizers [3,23,24,25] or for their anodophilic actuation behavior [26,27,28,29]. Different polymer–solvent combinations produce a unique mechanical response behavior which has not been determined for PVC-DBA gels, and DBA has been accepted as a promising plasticizer for PVC gels in DEAs [30]. The EPIC requires tensile constitutive models to describe the mechanical behavior of non-actuated gel segments. We note that PVC gels are highly viscoelastic based on their large liquid plasticizer content and preliminary analysis. Pure elastic and hyperelastic models are only strictly valid under steady-state conditions when the material has fully relaxed. Viscoelastic properties will be evaluated as part of a future study.

Secondary objectives are to develop a relationship between elastic modulus and plasticizer content and to validate isotropic and incompressibility assumptions. The linear elastic region provides a useful basis for correlating the elastic modulus with plasticizer content. This work demonstrates an empirical relationship through the power scaling law. The large liquid plasticizer content suggests that PVC gels are nearly incompressible and can be treated as incompressible materials to simplify modeling. This assumption is subjected to validation; however, it was found to not apply to highly plasticized gels at small strains. The reason is explored with polymer network analysis.

## 2. Elastic and Hyperelastic Constitutive Equations

### 2.1. Constitutive Equations

An elastic material is an object that returns to its undeformed configuration when the load is removed. It is defined within a region of strain appropriately named the elastic region. Linear elastic materials have a linear stress σ and strain ϵ relationship, known as the elastic modulus E, in the elastic region. It represents the sensitivity of stress due to strain. A linear relationship also exists between strains along the orthogonal axis. Poisson’s ratio v is transverse strain $\epsilon_{T}$ by axial strain $\epsilon_{A}$ or the strain in the direction of loading. Poisson’s ratio is a dimensionless quantity that only exists within the elastic region.

(1) $$E=\frac{\sigma }{\epsilon },v=-\frac{\epsilon_{T}}{\epsilon_{A}}$$

Power scaling laws have been applied to PVC gels for storage modulus, shear modulus, and elastic modulus [31,32,33]. In 1954, Walter showed the power law between elastic modulus and polymer volume concentration percentage [23]. We continue evaluating this relationship between the elastic modulus E and PVC volume concentration percentage c. A linear relationship is obtained when using logarithmic conversions, allowing for simple linear fitment. $E_{0}$ and η are the elastic modulus of pure PVC and the power law exponential, respectively.

(2) $$E=E_{0}c^{\eta }$$

(3) $$\log_{10}\left(E\right)=\eta \log_{10}\left(c\right)+\log_{10}\left(E_{0}\right)$$

Hyperelastic models approach large deformations using Helmholtz free energy Ψ, a thermodynamic potential expressing strain energy as a function of stretch and temperature. In an isothermal application, strain energy is expressed as a function of the deformation gradient $\Psi \left(F\right)$. To isolate deformation from rigid body motion, the deformation gradient is converted to the right Cauchy–Green deformation tensor and further to the right stretch tensor $C=F^{T}F=U^{2}$. Isotropic and homogenous materials under uniaxial loading have a diagonalized deformation gradient representing stretch under principle directions. $\lambda_{1}$ is axial stretch in the uniaxial loading and $\lambda_{2}$ and $\lambda_{3}$ are transverse stretches.

(4) $$F=\left[\begin{array}{ccc} \lambda_{1} & 0 & 0\\ 0 & \lambda_{2} & 0\\ 0 & 0 & \lambda_{3} \end{array}\right]$$

Since the deformation gradient is diagonalized, strain energy can simplify further to be a function of principle scalar invariants $\Psi \left(I_{1},I_{2},I_{3}\right)$. The first invariant $I_{1}$ represents isochoric total shape change through elongation. The second scalar invariant $I_{2}$ is the sum of principle minor determinants, encapsulating shear deformation and distortional effects. The third scalar invariant $I_{3}$ describes changes to volume or volumetric stretch.

(5) $$I_{1}=\lambda_{1}^{2}+\lambda_{2}^{2}+\lambda_{3}^{2}$$

(6) $$I_{2}=\lambda_{1}^{2}\lambda_{2}^{2}+\lambda_{2}^{2}\lambda_{3}^{2}+\lambda_{3}^{2}\lambda_{1}^{2}$$

(7) $$I_{3}=\lambda_{1}^{2}\lambda_{2}^{2}\lambda_{3}^{2}=J^{2}$$

Assumptions can be made to correlate the axial and transverse stretches. Isotropic materials exhibit equivalent transverse stretches, i.e., $\lambda_{2}=\lambda_{3}$. Incompressible materials exhibit no changes to volume. This makes the J=1, which is used in conjunction with Equation (7) to formalize a direct relationship between transverse and axial stretches. We presently treat PVC gels as isotropic and incompressible materials, which is subject to validation with Equation (8). It also reduces modified invariants to their standard form.

(8) $$\lambda_{2}=\frac{1}{\sqrt{\lambda_{1}}}$$

And this applies to the principle scalar invariants:

(9) $$I_{1}=\left(\lambda^{2}+\frac{2}{\lambda }\right)$$

(10) $$I_{2}=\left(2\lambda +\frac{1}{\lambda^{2}}\right)$$

(11) $$I_{3}=1$$

where λ is axial stretch $\lambda_{1}.$

Cauchy or true stress σ is the derivative of strain energy with respect to the scalar invariants, i.e., $\frac{\partial \psi }{\partial I}.$ The hyperelastic equations are expressed in incompressibility strain energy form and transformed to Cauchy stress in terms of axial stretch ratio λ using Equations (9)–(11). This allows direct measurements to be used in fitment.

The Neo–Hookean model (Equation (12)) is a simple hyperelastic model derived from statistical mechanics of Gaussian chains. It uses the shear modulus μ and bulk modulus k, if the material is compressible [34,35]. The linearity of the first invariant limits this model for large deformations and is preferentially used in small to moderate stretches, i.e., λ≈1.3. This means it cannot adequately capture strain hardening effects which may overpredict at lower stretches and underpredict at higher stretches. The lack of the second invariant tends to underpredict strain energy and therefore stress. The shear and bulk modulus can be determined prior to the model’s use, making it easier to implement.

(12) $$\psi \left(I_{1}\right)=\frac{\mu }{2}\left(I_{1}-3\right)\Rightarrow \sigma =\mu \left(\lambda^{2}-\frac{1}{\lambda }\right)$$

The Mooney–Rivlin model (Equation (13)) improves on the Neo–Hookean model by incorporating a linear dependence of the second invariant. This makes it more accurate up to axial stretches around 2.0, i.e., λ≈2.0; however, it may struggle to capture strain hardening in materials that deviate from the Gaussian chain theory. It is expected to fit the experimental data better than the Neo–Hookean model. The model fits stress–strain data to get the coefficient $C_{10},C_{01}$. These parameters balance the contributions of the first and second invariant [34,35]. Fitment is difficult to predict as the second coefficient $C_{01}$ can be made negative, leading to instabilities. This is especially true when the application is different from the fitted testing mode [35].

(13) $$\psi \left(I_{1},I_{2}\right)=C_{10}\left(I_{1}-3\right)+C_{01}\left(I_{2}-3\right)\Rightarrow \sigma =2\left(\lambda^{2}-\frac{1}{\lambda }\right)\left[C_{10}+\frac{C_{01}}{\lambda }\right]$$

The Yeoh model (Equation (14)) is a third order polynomial that depends on higher orders of the first invariant. It uses parameters $C_{10},C_{20},$ and $C_{30}$ as scaling powers to adjust contributions of each first invariant power. In contrast to the previous two models, the Yeoh model tends to accurately capture strain hardening due to its non-linearities. It is typically used in uniaxial test data up to 3.5 stretch, i.e., λ≈3.5. The lack of the second invariant $I_{2}$ means distortional deformations are not accounted and can potentially fail to accurately describe large shear and biaxial behaviors [34,35]. Modern modifications like the Power-Yeoh model can be used if the fitment is insufficient [36] but the Yeoh model tends to fit well to other testing modes.

(14) $$\psi \left(I_{1}\right)=C_{10}\left(I_{1}-3\right)+C_{20}{\left(I_{1}-3\right)}^{2}+C_{30}{\left(I_{1}-3\right)}^{3}\Rightarrow \;\sigma =2\left(\lambda^{2}-\frac{1}{\lambda }\right)\left[C_{10}+2C_{20}\left(I_{1}-3\right)+3C_{30}{\left(I_{1}-3\right)}^{2}\right]$$

The Gent model (Equation (15)) is a simple hyperelastic model built on modifying the Neo–Hookean model to incorporate strain hardening effects. It includes a non-dimensional parameter known as the locking parameter $J_{m}$ that limits chain extensibility in uniform networks to effectively capture sharp stress inflections [37] at large deformation, i.e., λ≈3.0. The Gent model also places more emphasis on the first invariant’s influence and uses shear modulus μ [34,35]. There is still no second invariant; therefore, distortional deformation may not be accurately described, but the first invariant is now non-linear. In addition, deviation from Gaussian chains and strain softening may lead to improper fitment. Despite these reservations, it is expected to fit well.

(15) $$\psi \left(I_{1}\right)=-J_{m}\frac{\mu }{2}\ln\left(1-\frac{I_{1}-3}{J_{m}}\right)\Rightarrow \sigma =\mu \left(\lambda^{2}-\frac{1}{\lambda }\right)\left[\frac{J_{m}}{J_{m}-\left(\lambda^{2}+\frac{2}{\lambda }-3\right)}\right]$$

The Ogden model (Equation (16)) is a popular highly flexible hyperelastic model. It uses stretch ratios directly rather than principle scalar invariants. As a result, the model cannot be constrained by the isotropic and incompressibility assumptions of Equation (8) and implemented in scalar invariants as in Equations (9)–(11). The Ogden model uses shear modulus terms μ with a dimensionless exponential α that controls stiffening along the uniaxial stretch direction [34,35]. This model can be used up to material failure. The high non-linearity of the model with only uniaxial testing data makes it prone to parameter non-uniqueness. Many parameter combinations can fit the uniaxial response but may fail to describe biaxial and shear loadings. The best fit in uniaxial data should be used with caution until another testing mode verifies uniqueness.

(16) $$\psi \left(\lambda_{1},\lambda_{2},\lambda_{3}\right)=\sum_{i=1}^{N}\frac{2\mu_{i}}{\alpha_{i}^{2}}\left(\lambda_{1}^{\alpha_{i}}+\lambda_{2}^{\alpha_{i}}+\lambda_{3}^{\alpha_{i}}-3\right)\Rightarrow \sigma =\sum_{i=1}^{3}\frac{2\mu_{i}}{\alpha_{i}}(\lambda^{\alpha_{i}}-\lambda^{-\frac{\alpha_{i}}{2}})$$

The extended tube model (Equation (17)) is one of the most physically realistic hyperelastic models based on statistical micro-mechanism models. It considers the cross-linking of the network, confining tube constraints, and volumetric deformations, creating a comprehensive model based around the molecular chains and their interactions. It is highly non-linear but uses higher orders of the first scalar invariant. The extended tube model uses four parameters: $G_{C}$ is the cross-linking contribution; $G_{e}$ is the constraint contribution; δ represents chain or tube constraints; β represents the rearrangement of crosslinks upon deformation [38]. Similar to the Ogden model, fitting with uniaxial data does not provide unique parameters. Different parameter combinations can reproduce the same uniaxial response.

(17) $$\sigma \left(I_{1},\lambda \right)=G_{C}\left(\lambda^{2}-\frac{1}{\lambda }\right)\left[\frac{1+\left(1+I_{1}^{2}-4I_{1}\right)\delta^{2}+\left(5I_{1}-I_{1}^{2}-6\right)\delta^{4}}{{\left[1-\left(I_{1}-3\right)\delta^{2}\right]}^{2}}\right]-\frac{2G_{e}}{\beta }\left[\lambda^{-\beta }-\lambda^{\frac{\beta }{2}}\right]$$

Engineering stress, or First Piola–Kirchhoff stress P, is measured in the experiment. It uses the undeformed area; however, large deformations have significant effects on the area. First Piola–Kirchhoff stress is related to Cauchy stress σ with the Jacobian and deformation gradient **F** (Equation (4)). The assumptions outlined in Equation (8) simplify the conversion; therefore, Cauchy stress is the First Piola–Kirchhoff stress scaled by axial stretch (Equation (18)).

(18) $$P=J\sigma F^{-T}\Rightarrow \sigma =P\lambda_{1}$$

### 2.2. Determination of Hyperelastic Material Parameters

Hyperelastic models, such as Neo–Hookean and Gent, use parameters attributed to mechanical properties. Many others have parameters that are more abstract, being used as smoothening or scaling factors. It is difficult to justify the interpretation of each model’s parameter when attempting to fit the optimal solution and tensile hyperelastic models for PVC-DBA gels have yet to be established in literature. To facilitate optimal parameters during fitment, the genetic algorithm is employed with the Levenberg–Marquardt algorithm. The genetic algorithm uses a random selection of parameters to analyze fitment against an objective function. The process is evolutionary with the best parameters of each iteration influencing the next generation. It is used to establish an initial guess for the Levenberg–Marquardt (LM) algorithm which is a damped least-squares algorithm popularly employed in non-linear fitting. This algorithm is influenced by its initial guess and can converge on a local minimum or suboptimal parameters. To minimize this, the genetic algorithm is performed five times with the normalized root-mean-squares as the objective function and a population size of 500. The average value for each parameter is passed to the LM algorithm as the initial guess.

## 3. Materials and Methods

### 3.1. EPIC Actuator

**Figure 1:**
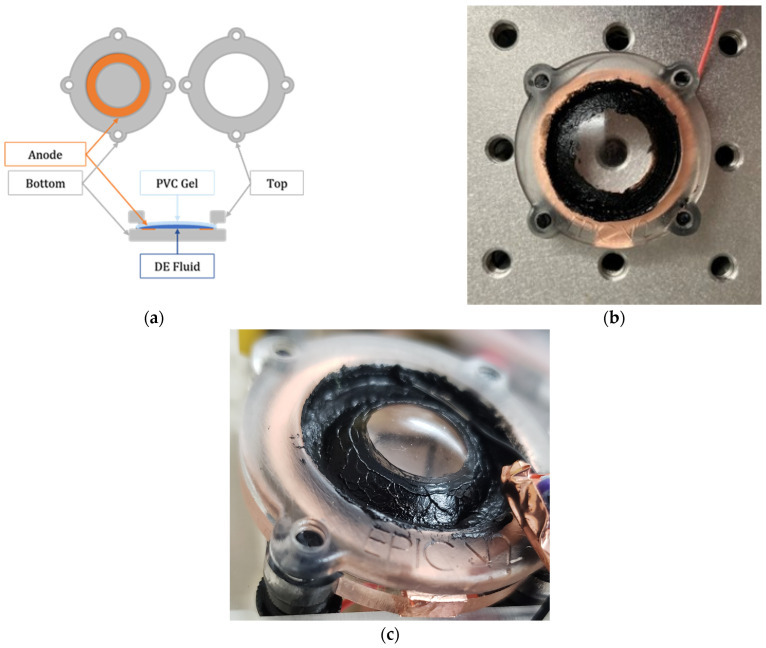
(**a**) A schematic diagram of the EPIC assembly, (**b**) an assembled EPIC ready for actuation, (**c**) the same EPIC with 3 kV applied.

The EPIC uses a rigid frame consisting of an acrylic substrate and resin printed cover plate. These are the bottoms and tops, respectively. They seal the PVC gel to encapsulate the dielectric fluid with the bottom substrate. An annular copper electrode fixed to the acrylic functions as the anode. The cover print incorporates a round opening to allow out-of-plane deformation during activation and integrated threads to secure the acrylic bottom plate. Figure 1 shows the actuator components in diagram form, a fully assembled EPIC, and the same EPIC actuated at 3 kV.

The PVC gel is first fixed into the frame. A dedicated injection port, integrated in the acrylic bottom plate, enables precise controlled dielectric fluid filling. Carbon grease applied to the gel surface directly above the anode serves as the cathode. An electric field of sufficient intensity facilitates actuation through anodophilic attraction of the gel. It begins with concentric attraction of the gel to the anode where the electric field intensity is the highest, which is the outer edge of the top plate opening. As voltage, and therefore electric field, increases, concentric attraction gradually occurs inwards radially. The gel exhibits spherical deformation by displaced dielectric fluid as shown in Figure 1c. Its magnitude is controlled by material properties and the applied voltage.

### 3.2. Material Preparation of PVC Gel Samples

PVC gels are optically transparent homogeneous materials. Owing to the plasticizer which pervades throughout the polymer network, a reduction in intermolecular interactions imparts flexibility and compliance. They are extraordinarily soft, possessing little to no flexural modulus. The degree of plasticization controls the gel’s network response by virtue of altering the magnitude of intermolecular interactions and depends on the type of plasticizer.

Polyvinyl chloride (PVC) resin, with a weight-average molecular weight $M_{W}$ = 233,000 g/mol and a number-average molecular weight $M_{n}$ = 99,000 g/mol, was used without further purification. The resin was dissolved in a mixed solvent system consisting of dibutyl adipate (DBA) and tetrahydrofuran (THF) and stirred for 24 h at 60 °C. The solvent-to-polymer ratio used was 30 mL THF per gram of PVC. All materials were sourced from Sigma-Aldrich Co. (St. Louis, MO, USA). PVC gels were prepared with various plasticizer-to-resin weight ratios denoted as PX where X represents the parts by weight of DBA per part PVC. Samples designated P2, P4, P6, and P8 correspond to DBA-to-PVC weight ratios of 2:1, 4:1, 6:1, and 8:1, respectively. Gels were cast in a loosely covered flat bottomed glass dish, curing at 22 °C for three weeks. Thickness across casts ranged from 2 to 3.75 mm.

### 3.3. Tensile Testing

A series of quasi-static tensile tests were performed to evaluate elastic and hyperelastic constitutive relationships. An Instron 5565 set the displacement rate and collected stress data while a camera system collected images for strain measurements through digital image processing. Ten independent datasets were collected for each plasticizer content. Of these datasets, five were selected for analysis in accordance with ASTM D638 [39], filtering outlier datasets and inconsistencies.

Quasi-static tensile testing was performed on an Instron 5565 Tensile Tester (Instron, Norwood, MA, USA) operating at monotonic displacement rate of 1 mm/min ± 0.01 mm and equipped with a 100 N ± 0.5% (of reading) load cell, collecting stress data. PVC gels were mounted with custom fixtures made of Form Labs BioMed Amber resin (Formlabs, Somerville, MA, USA). An integrated stop within the fixtures compressed the gels to 50 microns which prevented bursting when mounting. Following ASTM D638 standards, samples were cut into type-4 dog bone shapes with the gauge length shortened by 8 mm. This modification allowed the camera system to be positioned closer to the sample, increasing spatial-pixel resolution and measurable axial strains while decreasing test duration.

**Figure 2:**
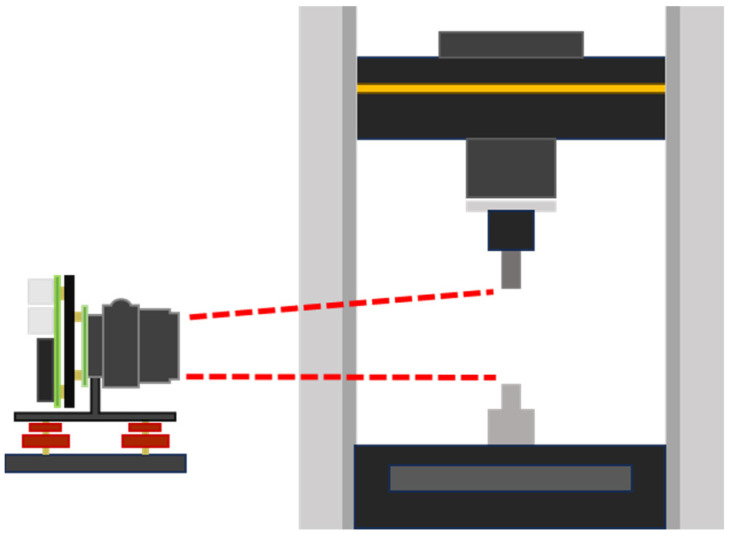
Diagram of the Instron 5565 and Raspberry Pi camera setup.

Strain was measured using a camera system that consisted of a Raspberry Pi 4 attached to the Pi HQ 12.3 MP camera module paired with Theia SL410 m lens. The camera was programmed in python and configured at 4 K resolution, ISO of 1 to reduce noise, and an exposure time of 31 ms to limit motion blur. The aperture was set based on lighting conditions. The camera was aligned within 0.5° of the Instron’s centerline then geometrically calibrated at 2 × zoom. This led to a wide FOV with a spatial-pixel resolution of 47.2 μm/pxl, which tailors to axial strain data collection. Stress data and images were sampled at 1 Hz. The setup is depicted in Figure 2.

**Figure 3:**
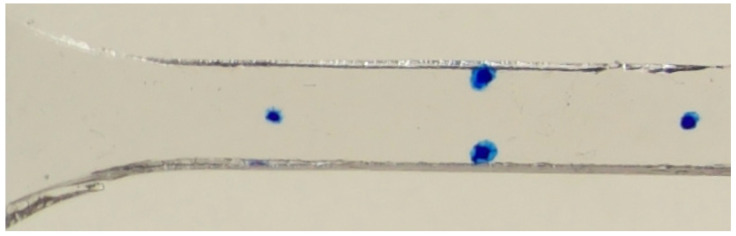
Image 156 of the fifth P2 dataset, showing the markers in the gauge length region.

Axial and transverse strains were determined using digital image processing to track marks on the gel surface as shown in Figure 3. Blue sharpie marks applied with a position template facilitated repeatable placement. For axial strains, two marks were placed 20 mm apart along the specimen’s centerline. Two more marks placed 6 mm apart along the midline determined transverse strains. The diameter of the marks is between 1 and 2.5 mm.

Thickness is the average of the four sharpie marks measured with Epsilon 1401-10 laser displacement sensor. Mitutoyo digital calipers measured the width.

## 4. Results and Discussion

### 4.1. Elastic Modeling

**Figure 4:**
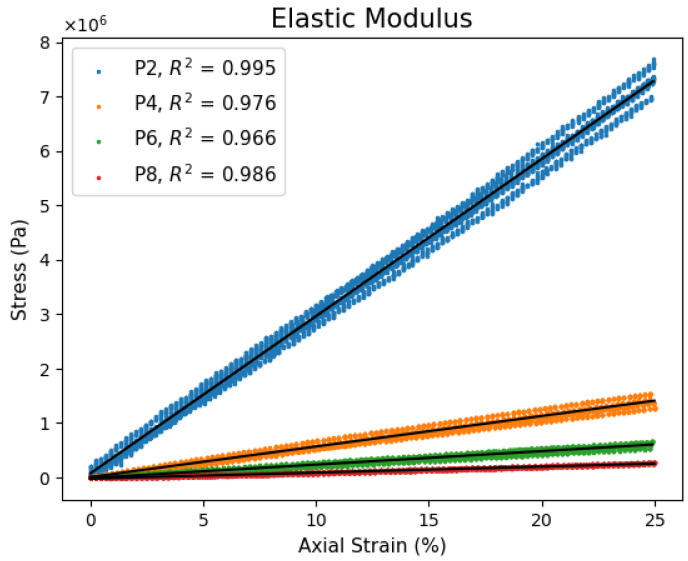
PVC gel elastic modulus for various plasticizer contents.

PVC gels are linearly elastic materials up to 25% strain under quasi-static conditions. Linear regression in this region attains coefficient of determinations above 0.966, which indicates high agreeability. Beyond 25% strain, gels show an upward stress inflection, indicating the beginning of strain hardening and thus establishing the limits of the linear elastic region. Figure 4 shows stress–strain data for P2, P4, P6, and P8 gels using five datasets each. Table 1 lists the average elastic modulus for each plasticizer’s content. The elastic modulus is inversely proportional to the plasticizer content, decreasing with increased plasticizer. These results and their magnitudes are consistent with Ali et al. [40] who performed elasticity measurements for P1, P2, P3, P5, P7, and P9 PVC-DBA gels. A gel that is loaded or strained can use Equation (1) to find its conjugated strain or stress once steady-state conditions have been met. As previously mentioned, linear elasticity does not consider viscoelastic time dependence, which is expected given that the gel bulk is majorly liquid plasticizer. Further studies on stress relaxation modulus and relaxation times will establish a time frame to which linear elasticity is valid.

Increasing the modulus as the plasticizer content decreases can be explained through the molecular structure of the gels. Gels were found to contain crystalline regions where polymer chain segments connect to physical crosslinks, creating a network spanning throughout the bulk that provides structure and rigidity to the gel [41,42,43]. The chains are coiled and entanglements in the amorphous regions promote interactions between crosslinks [44,45,46]. Deformation pulls the chain segments from their coiled configuration, which acts with entanglements and crosslinks in a restorative capacity. Gels with higher polymer concentrations readily entangle due to the smaller molecular spacing between chains [47]. The sequence length is also shortened between crosslinks [44,48]. The higher entanglement density reenforces the reactionary mechanical behavior by allowing for minimal chain extension in conjunction with enhancing junction site interactions. Decreasing the polymer concentration by increasing the plasticizer content increases molecular separation. The network becomes connected by fewer, longer chain sequences; therefore, less chains are capable of entangling, and the crosslinks are spatially further apart. The combination of fewer chains with less entanglement lowers the modulus as seen in the P8 gel.

**Figure 5:**
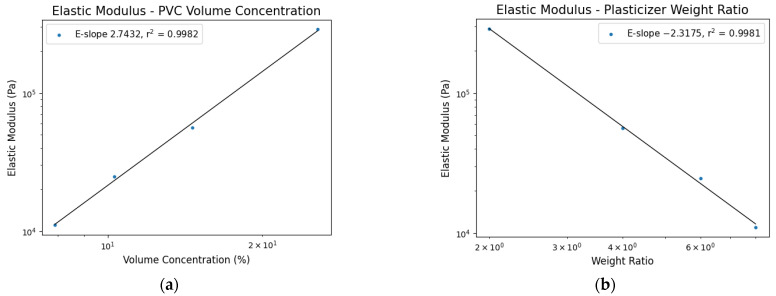
Elastic modulus related to PVC volume fraction concentration (**a**) and plasticizer weight ratio (**b**).

A correlation between the modulus and gel composition can be described by the power law. The elastic modulus versus PVC volume concentration percentage yields a linear relationship in log space with Figure 5a showing a fitting result. This relationship using Equation (2) is particularly useful because the elastic modulus $E_{0}$ of pure PVC can be readily determined. The calculated modulus of 11.84 MPa is close to 14 MPa in the previous study by Gent et al. [31] using a similar strain rate of 0.2%. As the polymer volume concentration approaches zero, the modulus vanishes since the polymer network provides the mechanical response characterized by the elastic modulus.

Using the power law provides an empirical relationship between material properties and polymer concentration. Li and Aoki have thoroughly applied power law to the quasi-equilibrium modulus and distance from the gel point (relative distance) [49,50,51,52,53]. Through the percolation theory, they have suggested that the strength of the gel is governed by the network sequence length and the networks are similar as PVC concentration changes post-gelation. From their work, we extrapolate the nature of the linear slope η as a description of the similar network structure in PVC-DBA gels; however, this only applies to PVC-DBA gels as solvent–polymer interactions and bonds affect the formation and alter structure.

PVC gels are typically characterized by their plasticizer weight ratio rather than by the PVC volume concentration. For convenience, the power–law relationship was applied using the plasticizer weight ratio to determine the corresponding elastic modulus (Equation (19)). As with the previous case, curve fitting revealed an additional linear trend as shown in Figure 5b. From any DBA-to-PVC plasticizer weight ratio X:1 the elastic modulus can be determined.

(19) $$E=1.4408•10^{6}{\left(X\right)}^{-2.3175}$$

It should be noted that this modulus vs. the plasticizer weight ratio should only be used within the fitment range. Low weight ratios like P0 approach infinity due to the negative exponential but higher ratios such as P1000 approach zero. This alternative formulation does not explicitly relate to the polymer network, but it is particularly useful for estimating the elastic modulus within the range from P2 to P8 weight ratios, providing guidance for tailoring mechanical properties of PVC gels in tensile applications.

**Figure 6:**
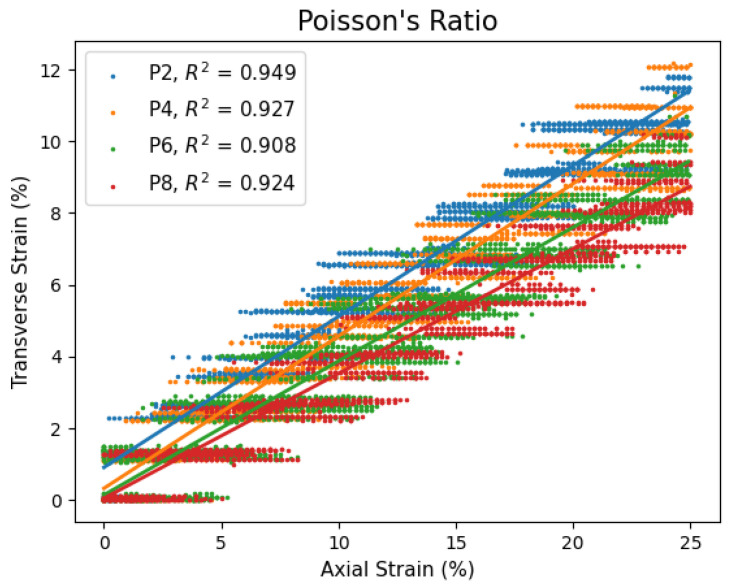
PVC gel Poisson’s ratio for various plasticizer contents.

Figure 6 presents transverse and axial strain data for five datasets of P2, P4, P6 and P8 gels in the established linear elastic region. Poisson’s ratio is the linear slope in this figure. Transverse strain data tended to a constant value, creating horizontal groupings. This is due to the camera resolution being insufficient to capture transverse deformation. As mentioned in the methods section, the camera system was tailored to measure axial strain. This led to a wide FOV with a spatial-pixel resolution of 47.2 μm/pxl. Changes in transverse strain were too small to be precisely recorded with this camera system. Despite this, all gels had decent linear fitments, considering the transverse strain data is discretized, with the lowest coefficient of determination being 0.908. An immediate trend can be observed with respect to the plasticizer content. The P2 and P4 gels had nearly the same slopes with a Poisson’s ratio of 0.42 and 0.43 but decreased with the P6 and further with the P8 to 0.37 and 0.35, respectively.

Poisson’s ratio for each gel is summarized in Table 2. PVC gels were initially believed to be nearly incompressible materials; therefore, Poisson’s ratio was anticipated to be near 0.49 and can be treated as incompressible. In addition, the variability of Poisson’s ratio with plasticizer content implies that the polymer network is not as well interconnected as previously thought. The deviation appears to be an intrinsic material behavior from the plasticizer’s influence on the polymer network.

The mechanical behavior of PVC gels originates from the polymer network and the interactions between crosslinks, either through direct chains connections or through entanglements in the amorphous regions [44,45,46,47,48]. The increase in plasticizer content reduces the polymer concentration, which lowers crosslink density, decreases entanglement density, and lengthens network chains between crosslinks. We propose that the observed Poisson’s ratio and its further decrease with increasing plasticizer content results from two coupled mechanisms: (1) chain slippage in the entanglements, which inhibits influence to surrounding crosslinks; (2) the straightening of longer chain segments, which delays load transmitting across the network.

**Figure 7:**
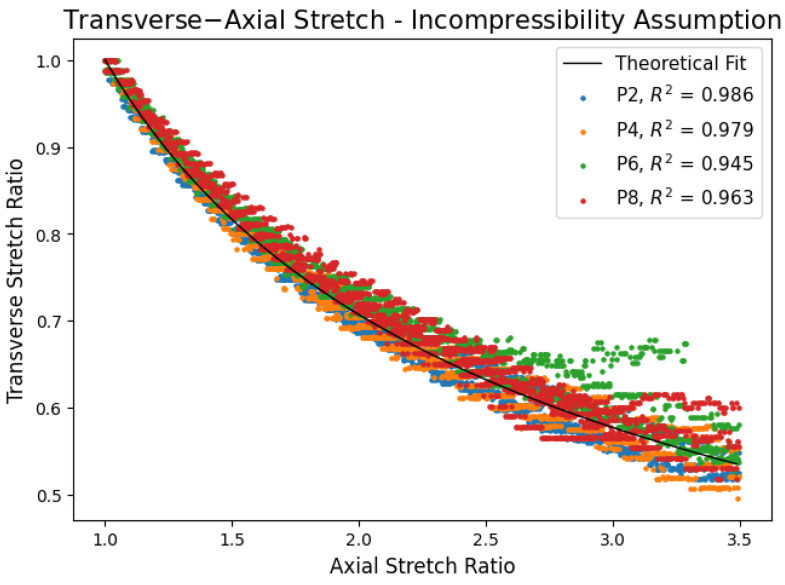
Validation of isotropic incompressibility conditions.

**Figure 8:**
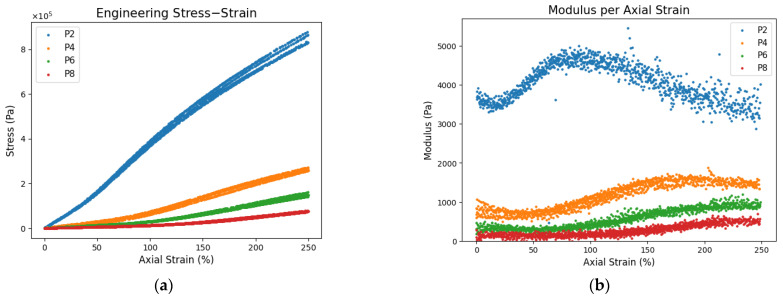
(**a**) Engineering stress–strain for all plasticizer contents and (**b**) modulus comparison showing the change in strain hardening.

This framework explains why highly plasticized P6 and P8 gels have lower transverse strains and thus Poisson’s ratios. The network is relatively sparse with greater molecular mobility and chain extensibility that effectively hinders transverse deformations. In contrast, P2 and P4 gels form dense, tightly interconnected networks with higher entanglement densities. These networks are less prone to slippage, and their shorter network chains engage more readily, resulting in a stronger transverse strain response; however, the existence of chain slippage and lengthening still reduces the transverse strain; therefore, the gels are not quite nearly incompressible. These conclusions are supported with isotropic and incompressibility predictions from the continuum mechanics approach (Equation (8)) shown in Figure 7 and are further supported by large strain stress data presented in Figure 8.

Figure 7 shows experimental transverse stretch plotted against axial stretch and compared to theoretical transverse stretch predictions of isotropic and incompressible materials using Equation (8). All the gels had a coefficient of determination above 0.945, which indicates high agreement with theoretical predictions. Experimental transverse stretches appear discretized due to the same camera resolution inadequacy explained in the prior section. A P6 and P8 gel dataset showed odd deviation due to lighting changes later in the experiments. Efforts were made to minimize the deviation.

The agreement in Figure 7 indicates PVC gels can generally be treated as isotropic and incompressible materials; however, the P6 and P8 gels deviate slightly from theoretical predictions. For any given axial stretch, the measured transverse stretches were offset above the theoretical curve, with P8 showing the largest deviation. This corresponds to smaller transverse strains. Interestingly, both P6 and P8 gels follow the theoretical curve shape despite the offset, implying that a delayed transverse response occurred at small stretches. This behavior is consistent with the lower Poisson’s ratios observed for the P6 and P8 gels.

The P8 gel, having the lowest polymer concentration, exhibits the lowest entanglement density and highest chain extensibility. At small deformations, the chains primarily slip within the entanglements and uncoil. The sparce network in conjunction with weakly interconnected crosslinks results in a reduced network response, making transverse deformation small, which is the observed offset in Figure 7. At larger strains, network chains are pulled taut, and slippage diminishes, making the network response stronger. As a result, the P8 and P6 gels follow the theoretical curve although offset. As polymer concentration increases, higher entanglement densities with shorter chain sequences strengthen the network response further. The P2 and P4 gels follow theoretical predictions quite well and do not exhibit the offset as the P6 and P8 gels. This is attributed to enhanced entanglement density and shorter network chains producing more immediate network responses.

From the continuum mechanics perspective, PVC-DBA gels can be considered isotropic and incompressible, albeit appreciable deviation occurs in highly plasticized gels and at small strains where Poisson’s ratio deviates from nearly incompressible or incompressible conditions, i.e., v≈0.49–0.5. At low strains in the linear elastic region, slippage and uncoiling reduces the mechanical response behavior causing the observed lower Poisson’s ratio. At higher strains, when these mechanisms cease, the gels act as isotropic and incompressible materials. This can be validated when showing strain hardening in Figure 8.

While the stress–strain curves of the gels show linearity in small strain range as shown in Figure 4, they exhibit non-linear mechanical behaviors at large strains as shown in Figure 8a. Non-linear behavior is a result of strain hardening, the point at which chains have reached finite extensibility; therefore, the response is dominated by entanglements and crosslinks resisting unravelment, and strain softening, the points where they begin unraveling. By strain hardening, many chains are aligned and uncoiled, producing a characteristic increase in stress response. After sufficient deformation, their stress response decreases, indicating chain separation from entanglements or ruptured crosslinks.

Strain hardening occurs in P2 gels around 30% strain. This is seen in Figure 8b where the change in modulus begins to increase. It occurs later in P4, P6, and P8 gels around 40%, 60% and 110% strain, respectively. At the same time, the strain hardening regime is longer with smaller modulus change. This suggests the network chains in the P2 gel are much shorter and slip minimally. The network is more responsive from the interconnectedness of the entanglements; therefore, the mechanical response in the strain hardening regime is a change in modulus around 1500 Pa. As the plasticizer content increases, the entanglements weaken due to slippage and chains need more elongation before having the same influence. The overall network response is ultimately reduced, which is apparent in the P8 gel. This gel had a modulus change of around 300 Pa which indicates a lack of interconnectedness. This, with the isotropic and incompressible assumptions, justify the reasonings behind the decrease in elastic modulus and Poisson’s ratio with increasing plasticizer content. It stems from entanglement interactions which depend on the polymer concentration. Gels with high polymer concentrations have strong networks with higher entanglement densities, low slippage, and small network links. This makes the material more responsive through its interconnectedness. Decreasing the polymer concentration reduces the network strength and interconnectedness; therefore, it has weaker mechanical behavior.

### 4.2. Hyperelastic Modeling

**Figure 9:**
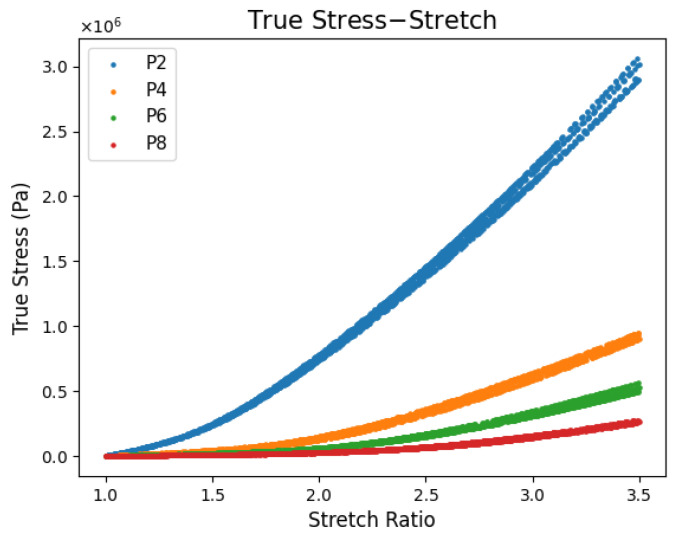
True stress–stretch relationship for each plasticizer content.

Figure 9 presents the true stress–stretch relationship for five datasets at each plasticizer content. Stretch is used rather than strain as hyperelastic models are derived in terms of principle stretches. Engineering stress is converted to true stress using Equation (18). To improve figure readability, every 25th data point is plotted while full datasets are used in curve fitting. All samples were stretched until failure, which occurred around 3.5–4 stretches. Since PVC gels are not expected to stretch beyond 3.5 (250% strain) in the EPIC, this was selected as upper bound for fitment.

We note that fitting simpler models such as the Neo–Hookean will be disenfranchised as a result. Their simplicity cannot sufficiently capture non-linearities that arise from strain hardening and strain softening. It is within the scope of this experiment to propose a material model that adequately captures the anticipated stretch range of PVC gels in the EPIC and not determine optimal parameters for each hyperelastic model in their typical stretch fitment regime.

**Figure 10:**
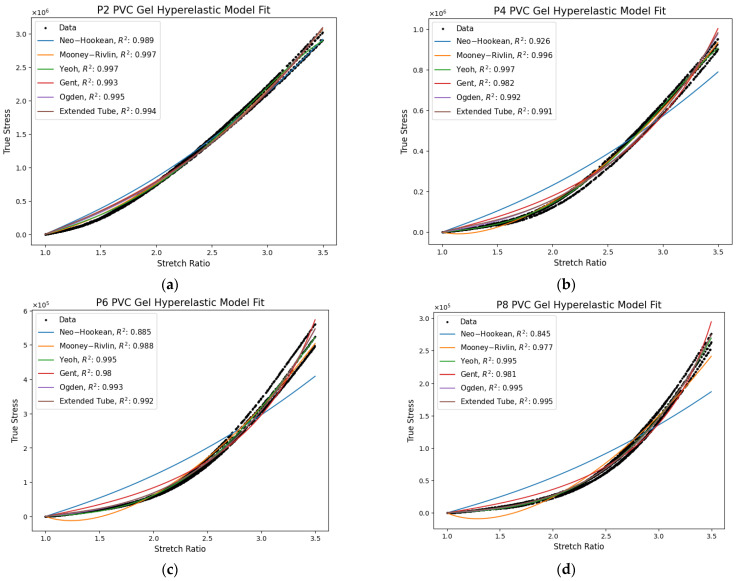
Hyperelastic fitting for (**a**) P2, (**b**) P4, (**c**) P6, and (**d**) P8 plasticizer contents.

Figure 10 shows the optimal hyperelastic model fitments evaluated up to 3.5 stretch, using the corresponding equations in Section 2.1. Table 3 presents the normalized root-mean-square-error (NRMSE) normalized by the average stress value of each gel dataset. The models evaluated in this study are some of the most popular but have yet to be evaluated for PVC-DBA gels. Simpler models such as Neo–Hookean, Mooney–Rivlin, and Gent struggled to capture the material response with P4, P6, and P8 gels, as indicated by the lower coefficient of determination and higher NRMSE. These models were sufficient for P2 gels, which behave most like a Gaussian chain network whose theory is the foundation of these models. The Yeoh, Ogden, and extended tube models performed well for all gels, as noted by their high coefficient of determination and NRMSE being less than 10%. The universality of each comes from higher-order dependence on the invariants or the approach taken for their derivation.

The Neo–Hookean model has a linear dependence on the first invariant for its strain energy function as stated in Equation (12). This prevents it from capturing strain hardening and softening at large deformations for the P4, P6, and P8 gels. It is derived from statistical mechanics of Gaussian chains, which does not account for entanglement slippage and assumes chain lengths are fixed. P2 gels are closest to Gaussian chains; hence, the model fits well up to 3.5 stretch. Correspondingly, the NRMSE increases dramatically from 8.4% up to 37.6% from P2 to P8, respectively.

The Mooney–Rivlin model in Equation (13) follows suite in linearity for the first and second invariant in its strain energy function, but it is also unable to capture strain stiffening for P4, P6, and P8 gels. While NRMSE is smaller than the Neo–Hookean, reaching 14.35 for P8 gels, the genetic algorithm favored data after hardening; therefore, small stretch data was grossly underpredicted. As a result, the second coefficient $C_{01}$ was negative, leading to the prediction of the compressive stress response, which is impossible.

The Yeoh model provides the best fitments up to 3.5 stretch, as indicated with having the highest coefficient of determination and lowest NRMSE of all models for all gel samples. Its higher-order dependence on the first invariant in the energy function, as shown in Equation (14), captures strain hardening and softening. While the second invariant is not included, the implementation of isotropic and incompressibility constraints into the first invariant adds some consideration of distortional deformation. It should be noted that using this model for pure shear may inadequately encompass the mechanical response. The model can also extrapolate poorly outside the fitted stretch range due to polynomial scaling. This is seen in the P2 gel curving downwards at high stretches.

The Gent model performed better than the Neo–Hookean but slightly worse than the Mooney–Rivlin model. It fits well for the P2 gel, having 6.63% NRMSE, but its curve did not accurately follow the experimental data. It uses the natural logarithm of first invariant (Equation (15)) and a parameter to limit chain extensibility. It assumes a uniform network with finite extensibility whereas the P4, P6 and P8 gels have more chain mobility through slippage and weaker junction sites. This is not featured in the locking parameter [37]. As a result, the model appreciably follows the experimental data as shown in Figure 10, attaining 12.24 to 13.3% NRMSE for P4 to P8, respectively. In these fitments, the curve concludes with a visible upwards inflection at large stretches.

The Ogden model and extended tube models consistently achieved high coefficients of determination, i.e., $r^{2}\approx 0.993\pm 0.003$ and NRMSE less than 10%, indicating high levels of fitment. These models are derived differently from the others, using direct stretch ratios and statistical micro-mechanism theory, respectively. They do not consider the isotropic and incompressibility constraints. For these reasons, the models are the least acceptable outside uniaxial data and require additional testing. Each model is highly non-linear with 6 and 4 parameters, respectively. The optimal parameters for each model and plasticizer content changed dramatically across several fitment iterations. For example, it is expected to have variable exponential signs in the Ogden model to account for strain hardening and softening. In a few convergences, they were all positive with a single negative shear term. The extended tube model followed suite with the tube constraint parameter δ being negative or zero and the crosslink rearrangement parameter β being higher in the P2 gel rather than the P8. This is a consequence of parameter non-uniqueness. Additional data in biaxial or shear is needed to facilitate accurate distortion modeling.

Of the models, the Yeoh model is proposed for use in describing the hyperelastic mechanical behavior in PVC gels up to 3.5 stretch. The higher-order dependence on the first invariant sufficiently captures axial deformation. It may not accurately describe pure shear; however, the EPIC uses PVC gels around 0.5 mm thick so that thin elastic shell assumptions can be utilized. In addition, this model does not suffer from non-uniqueness as in the Ogden and extended tube models while maintaining simplicity. This latter point helps in solution convergence for Multiphysics modeling. The parameters for the Yeoh model are listed in Table 4 for each gel with the corresponding coefficient of determination. Equation (20) provides an example of Cauchy stress of the Yeoh model with optimal parameters. Using Equation (18), it can be converted to engineering stress.

(20) $$\sigma =2\left(\lambda^{2}-\frac{1}{\lambda }\right)\left[85,622+2•\left(5800\right)\left(I_{1}-3\right)+3•\left(-272\right){\left(I_{1}-3\right)}^{2}\right]$$

## 5. Conclusions

In the present study, elastic and hyperelastic constitutive tensile relationships of PVC gels with varying DBA content were analyzed. A linear elastic region was identified up to 25% strain. The elastic modulus and Poisson’s ratio were measured using this strain regime. Both material properties were shown to decrease with increasing plasticizer content with elastic modulus decreasing from 288.8 kPa to 11 kPa and Poisson’s ratio from 0.42 to 0.35. We propose that the plasticizer content influences the intermolecular connectiveness of the network, adversely affecting the mechanical response. Low plasticizer contents allow for tight networks with many entanglements that limit chain slippage. This results in a more immediate influence on surrounding chains for a larger stress response and transverse strain. The increasing plasticizer content promotes a weakly connected network which reduces the elastic modulus and Poisson’s ratio. P2 and P4 gels can be treated as isotropic and incompressible materials with appreciable errors at small strains; however, this becomes increasingly less valid for P6 and P8 gels. Their weakly interconnected networks inhibit transverse strains because of mechanisms such as slippage and longer chain extensibility in a weakly entangled network. For high strains, all gels can be treated as isotropic and incompressible. A relationship between plasticizer content and elastic modulus was determined through the power scaling law.

Through analyzing several hyperelastic models, with emphasis on determining ideal initial guesses using the genetic algorithm, the Yeoh model is suggested to be used in hyperelastic tensile applications of the EPIC. It accurately captured strain hardening and softening up to 3.5 stretch, possessing a maximum NRMSE of 6.85%; however, its lack of the second invariant may limit the accuracy of distortions. Together, these findings establish a constitutive basis for PVC gels with DBA plasticizer, incorporating small strain elasticity, large strain non-linear behavior, and network analysis while providing suggestive insight into the network structure required for accurately modeling the EPIC.

This study can be improved by using a camera system with higher spatial-pixel resolution. The current system, which uses a 12.3 MP sensor, achieves a resolution of about 47.2 μm/pxl. While this is sufficient for axial strain measurements, it is insufficient for the smaller changes exhibited in transverse strain data. The limited resolution caused transverse data discretization, which introduces uncertainties for Poisson’s ratio measurements.

This present work focuses primarily on steady tensile behaviors in PVC gels, treating the material response as time-independent once stress relaxation ceases. Gels contain a large fraction of liquid plasticizer, and the polymer chains are susceptible to mobility. These features provide grounds for time-dependent responses that facilitate transient material behaviors. Future studies should aim to evaluate gels within a viscoelastic perspective, analyzing stress relaxation and dynamic material motion to capture transient behavior, which are required to characterize the EPIC in motion.

## Figures and Tables

**Table 1:** Elastic modulus for each plasticizer content up to 25% strain.

Plasticizer Ratio	Elastic Modulus (Pa)
P2	288,751
P4	56,058
P6	24,707
P8	11,047

**Table 2:** Poisson’s ratio for various plasticizer contents.

Plasticizer Ratio	Poisson’s Ratio
P2	0.42
P4	0.43
P6	0.37
P8	0.35

**Table 3:** Normalized root-mean-square error for each model in percentage.

Plasticizer Ratio	Neo–Hookean	Mooney– Rivlin	Yeoh	Gent	Ogden	Extended Tube
P2	8.4	4.53	4.15	6.63	5.73	6.32
P4	24.71	6.01	4.9	12.24	7.97	8.58
P6	31.48	10.13	6.85	13.18	8.03	8.21
P8	37.58	14.35	6.63	13.3	6.88	6.9

**Table 4:** Yeoh parameters and coefficient of determination for each plasticizer content.

Plasticizer Ratio	Coefficient of Determination	$\left[C_{10},C_{20},C_{30}\right]$
P2	0.9972	[85,622, 5800, −272]
P4	0.9971	[9061, 3175, −116]
P6	0.9946	[3106, 1545, −40]
P8	0.9952	[1365, 593, −5.99]

## Data Availability

The original contributions presented in this study are included in the article. Further inquiries can be directed at the corresponding author.

## Abbreviations

The following abbreviations are used in this manuscript:

PVC	Polyvinyl chloride
PX	Plasticizer to PVC weight ratio (X:1)
DBA	Dibutyl adipate plasticizer
THF	Tetrahydrofuran
DEA	Dielectric elastomer actuator
EPIC	Electrohydraulic actuator powered by induced interfacial charges
HASEL	Hydraulically amplified self-healing electrostatic
DE	Dielectric
KAIST	Korea Advanced Institute of Science & Technology
BOPP	Biaxially oriented polypropylene
LM	Levenberg–Marquardt
NRMSE	Normalized root-mean-square-error
